# Supplementary material for: MEK inhibitors block growth of lung tumours with mutations in ataxia–telangiectasia mutated
Source: Nat Commun. 2016 Dec 6;7:13701. doi: 10.1038/ncomms13701 (PMC5150652; doi:10.1038/ncomms13701)
Supplement: Supplementary Information — Supplementary Figures 1-13 and Supplementary Tables 1-5 [file ncomms13701-s1.pdf]

Supplementary Figure 1

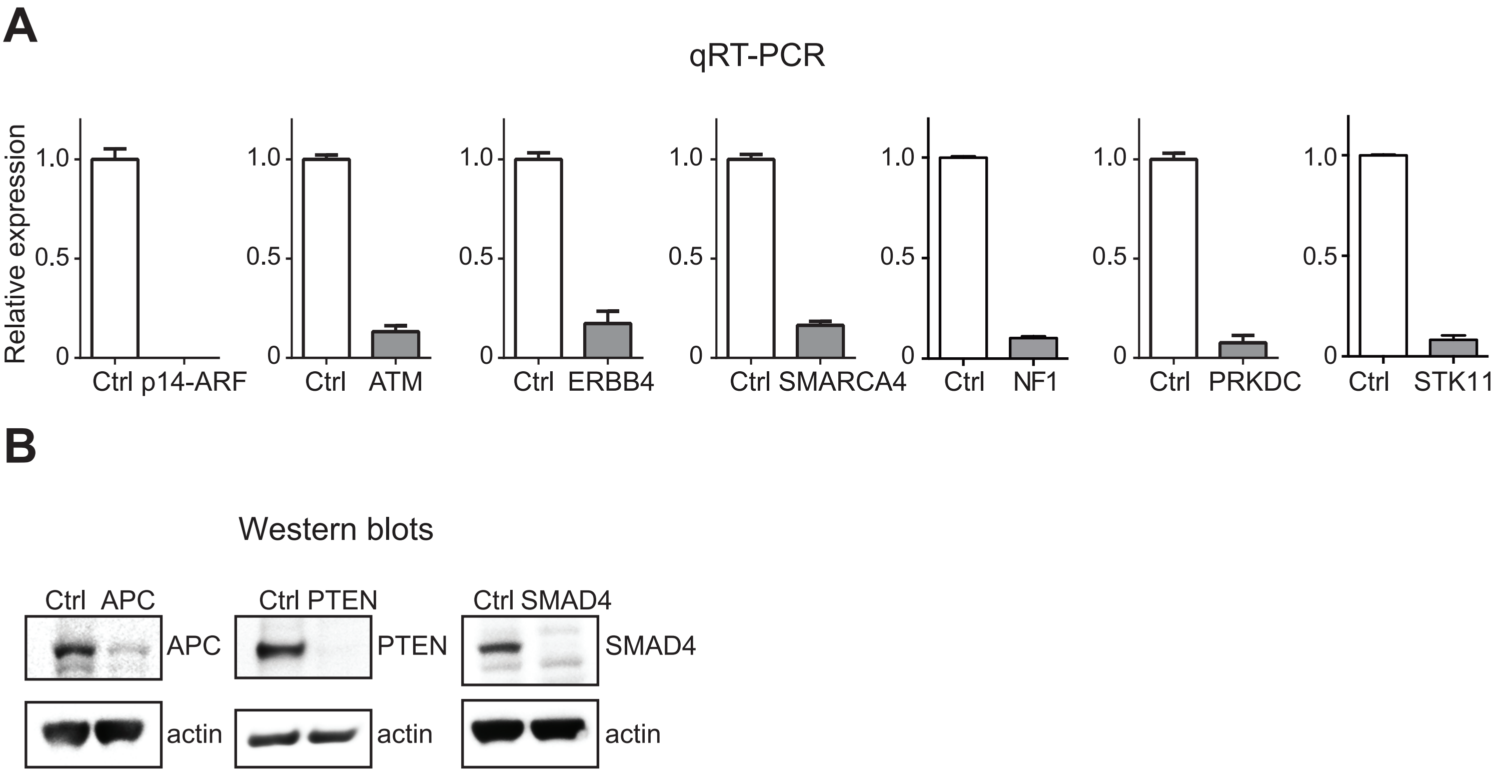

Supplementary Figure 1. Validation of vectors used for isogenic cell line panel. **(A)** mRNA expression analysis of indicated knockdown vectors in AALE cells by quantitative real time PCR. GAPDH was used as the reference gene. Error bars indicate standard deviation (n=3). **(B)** Western blot analysis of AALE cells infected with indicated shRNA vectors.

Supplementary Figure 2

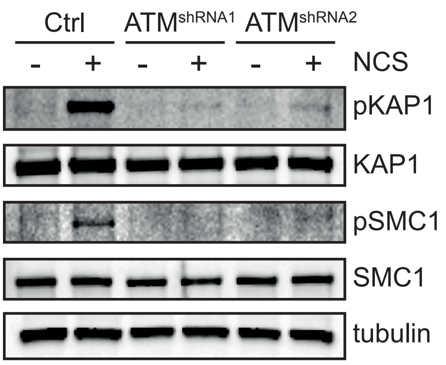

Supplementary Figure 2. Characterization of ATM knockdown in AALE cells. Western blot analysis of AALE cells infected with indicated vectors for pKAP1 and pSMC1 in response to treatment with neo-carzinostatin (50 ng/ml, 30 minutes).

Supplementary Figure 3

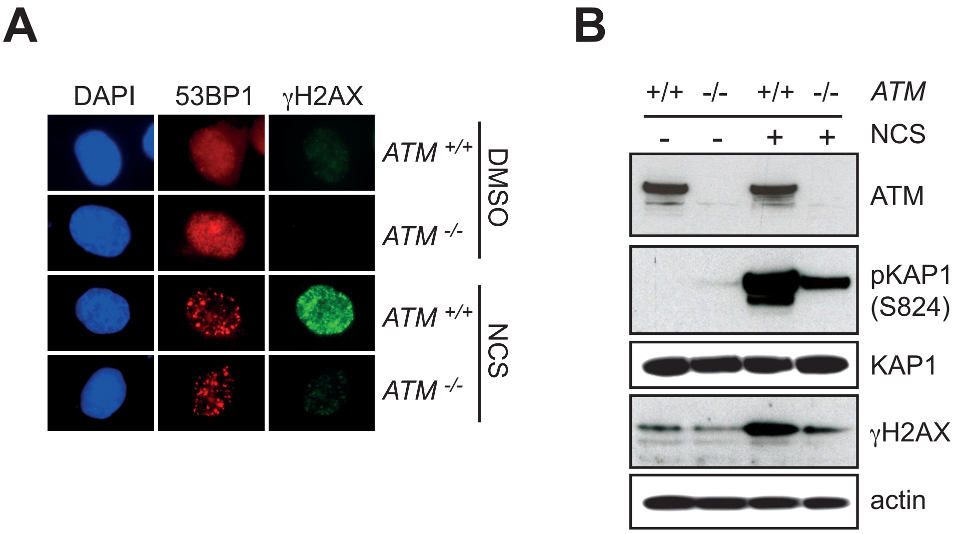

Supplementary Figure 3. Validation of ATM inactivation. (A) Immunofluorescence microscopy analysis of ATM knockout and control NCI-H322 cells for 53BP1 and phosphorylated H2AX (γH2AX; S139) treated with neocarzinostatin (50 ng/ml, 30 minutes). **(B)** Western blot analysis of NCI-H322 cells as in (A).

Supplementary Figure 4

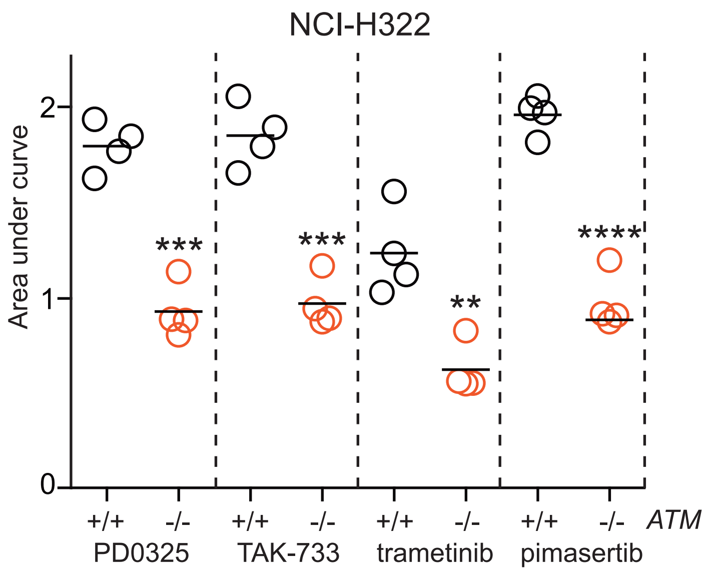

Supplementary Figure 4. ATM inactivation using CRISPR/Cas9 engineering renders lung cancer cell lines sensitive to MEK inhibition. Area under curve (AUC) of dose-response experiment of NCI-H322 cells in which both ATM alleles have been inactivated (ATM-/-) or unedited control (ATM+/+) treated with depicted compounds for 5 days. Each circle represents an independent clone. Data is normalized to vehicle treated cells. ** P < 0.01, *** P < 0.001, **** P < 0.0001, two sided t test.

Supplementary Figure 5

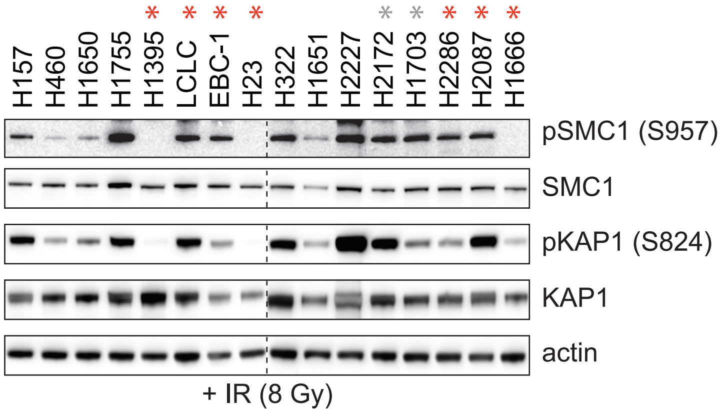

Supplementary Figure 5. DNA damage response of ATM mutant lung cancer cell lines. Western blot analysis of indicated lung cancer cell lines treated with ionizing radiation (8 Gy). Red asterisks indicate cell lines with confirmed ATM mutations, grey asterisks indicate cell lines with presumably neutral mutations (PolyPhen).

Supplementary Figure 6 (I)

A

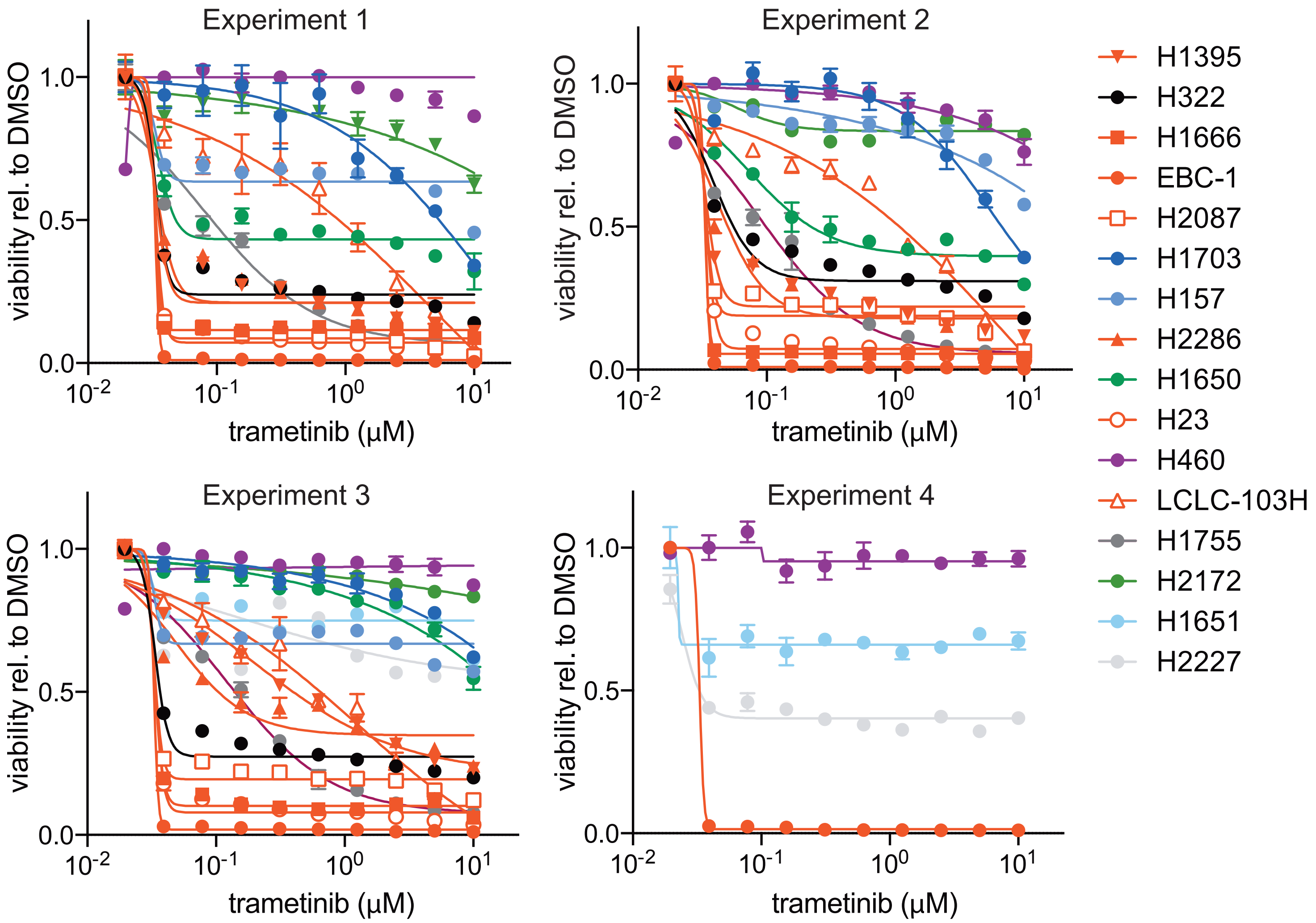

B

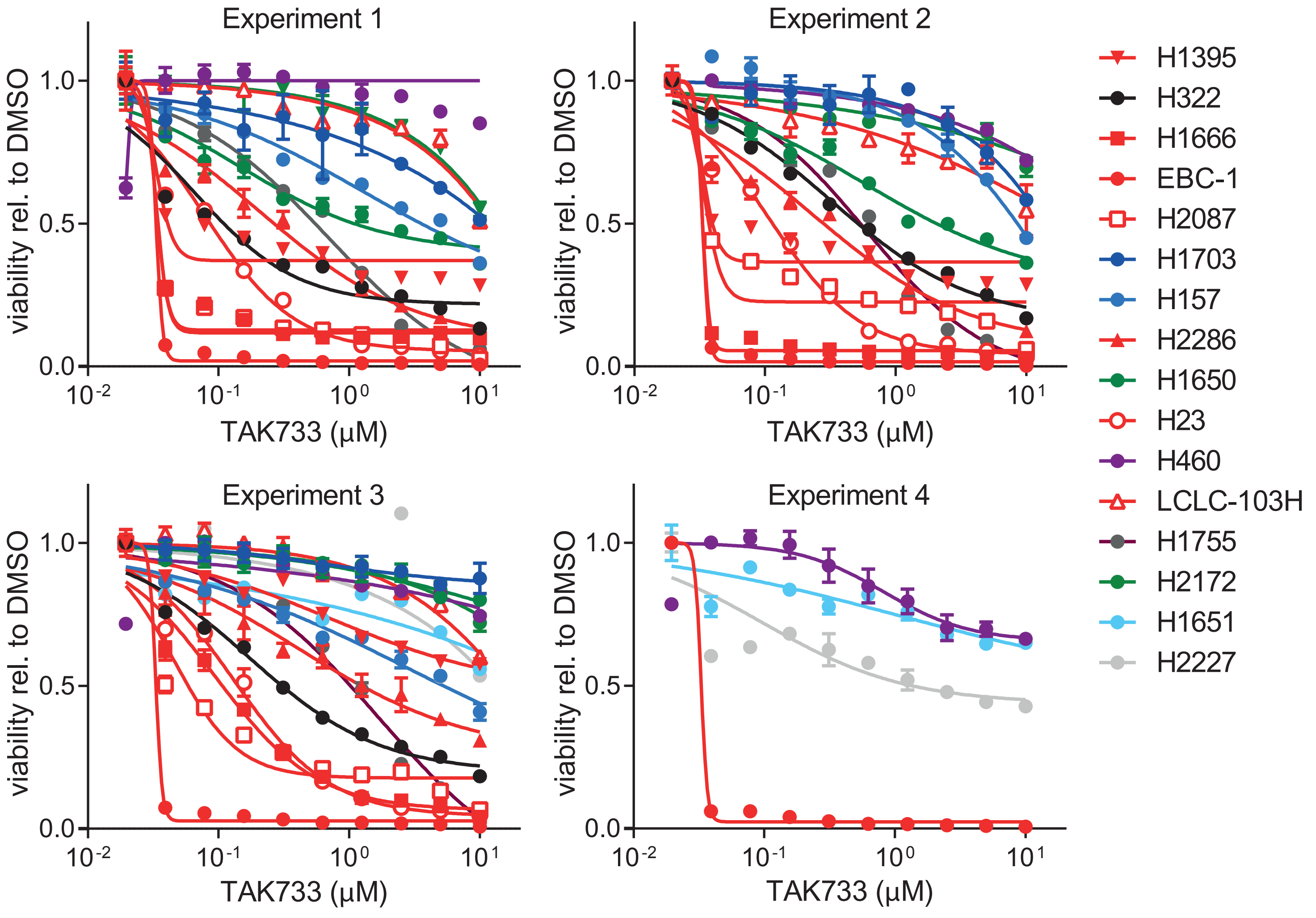

Supplementary Figure 6 (II)

Supplementary Figure 6. Response of lung cancer cell lines to MEK inhibitors. (A, B) Individual dose-response experiments for 16 lung cancer cell lines treated with trametinib (A) or TAK733 (B). Data was normalized to vehicle treated cells and error bars indicate standard deviation derived from replicate drug treatments (n = 3). Cell lines with damaging ATM mutations (PolyPhen) are labeled in red.

Supplementary Figure 7

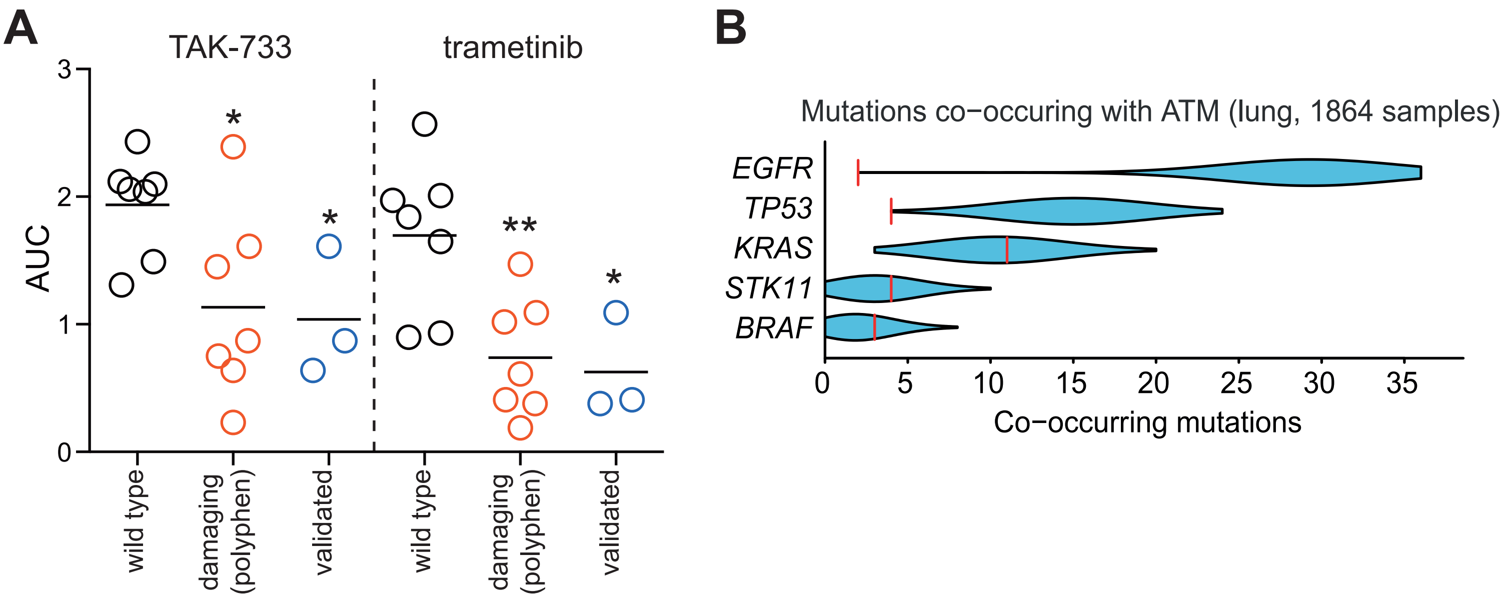

Supplementary Figure 7. Enrichment of validated ATM mutant cells among sensitive lines. (A) AUC data from 16 cell lines as in (Fig. 3B) for trametinib and TAK-733. Functionality of ATM mutations is indicated. Neutral mutations (NCI-H1703, NCI-H2172) were not included in the analysis. Black bars indicate mean, * $P < 0.05$, ** $P < 0.01$, two sided t test. **(B)** Violin plots showing distribution of simulated co-occuring mutations for ATM with EGFR, TP53, KRAS, STK11 or BRAF based on COSMIC data. Red lines indicate observed co-occurance for each combination.

Supplementary Figure 8

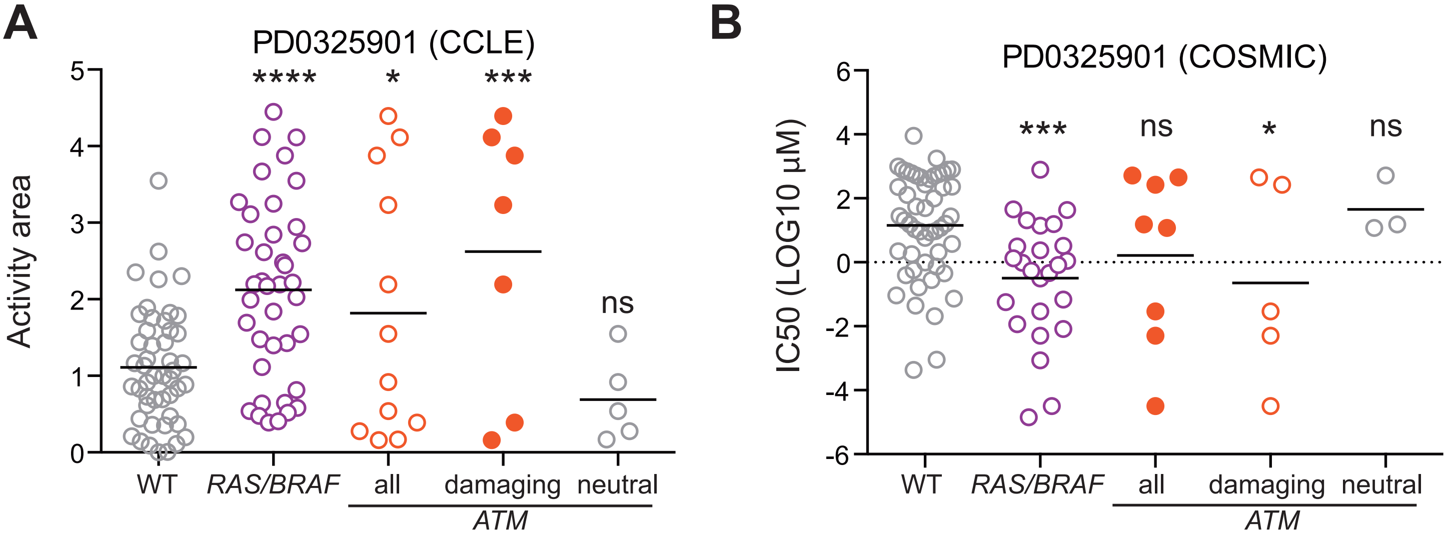

Supplementary Figure 8. Sensitivity of lung cancer cell lines in the Cancer Cell Line Encyclopedia (CCLE) and COSMIC database to the MEK inhibitor PD0325901. (A) High Activity Area Score (AAS) indicates drug sensitivity. Each circle indicates a single cell line and cell lines are grouped according to genotype (WT = wild type for K-Ras, H-Ras, N-Ras, BRAF, c-RAF and ATM; ATM = ATM mutant; RAS = K-Ras, H-Ras or N-Ras mutant). ATM mutations are labeled according to PolyPhen (damaging >0.9, neutral <0.9). Black bar indicates mean AAS. * P <0.05, *** P < 0.001, **** P <0.0001, ns = not significant, two sided t test. **(B)** Inhibitory concentration (IC) 50 of lung cancer cell lines in the COSMIC/Sanger dataset. Each circle indicates a single cell line and cell lines are grouped according to genotype as in (A). ATM mutations are labeled according to PolyPhen (damaging >0.9, neutral <0.9). Black bar indicates mean IC50. * P < 0.05, *** P <0.001, ns = not significant, two sided t test.

Supplementary Figure 9

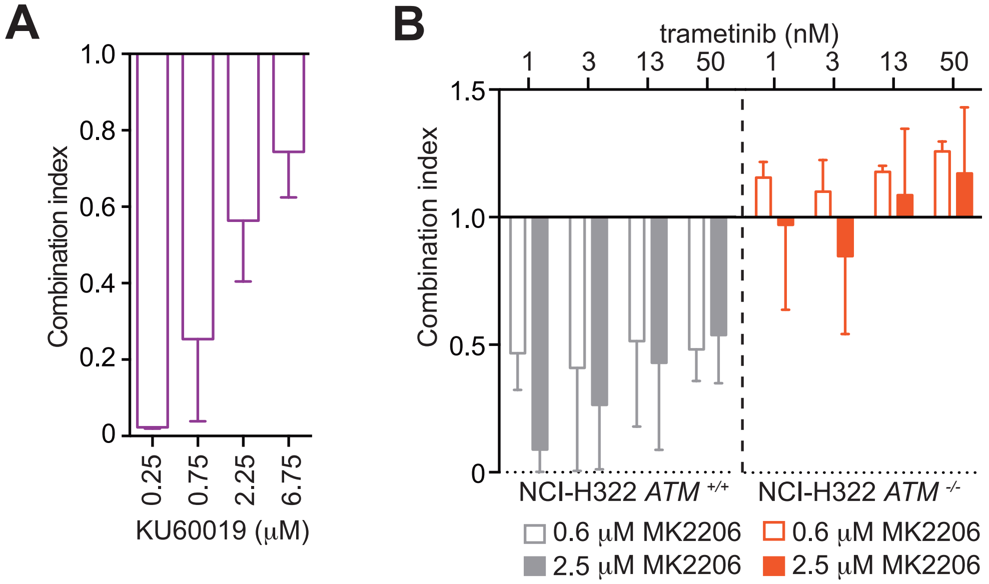

Supplementary Figure 9. Drug synergy experiment of MEK inhibition combined with ATM or AKT inhibition. (A) Drug synergy in AALE cells with ATM inhibitor (KU60019) and trametinib (40 nM). Chou Talalay combination index calculated from experiment in figure 5A is shown. Error bars indicate standard deviation (n = 3). **(B)** Chou Talalay combination index derived from drug synergy experiment on NCI-H322 control (+/+, grey bars) and ATM knockout (-/-, red bars) cells treated with trametinib and AKT inhibitor MK2206 (0.6 μM and 2.5 μM) shown in 5E. Error bars indicate standard deviation (n = 3).

Supplementary Figure 10

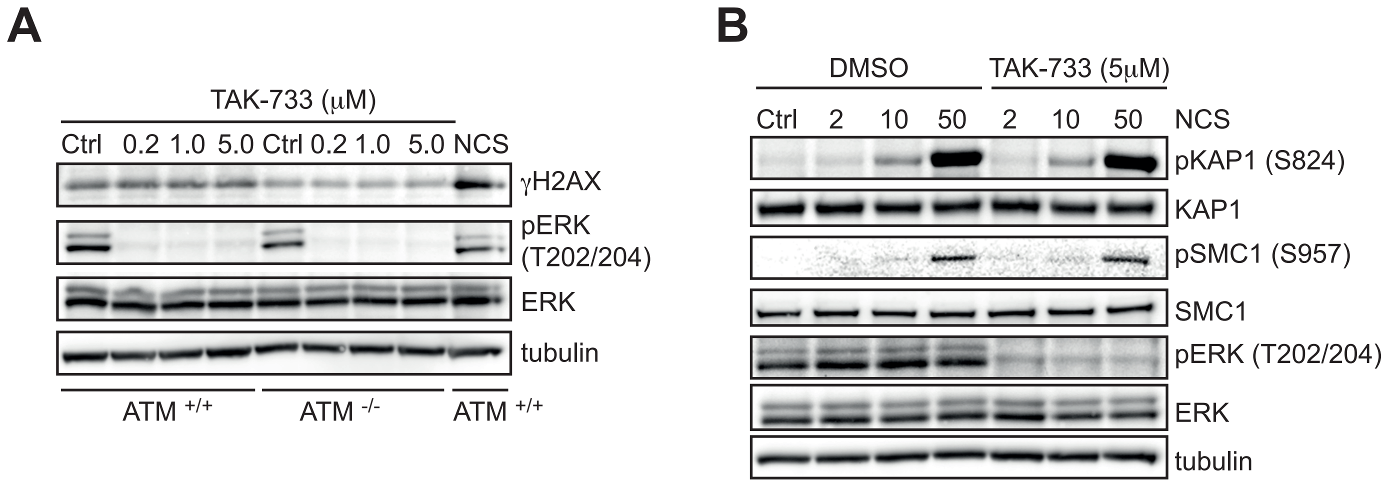

Supplementary Figure 10. MEK inhibition does not affect the DNA damage response. (A) Western blot analysis of wild type or ATM mutant NCI-H322 cells treated with TAK-733 (6 hours) or NCS (50 ng/ml, 30 minutes). **(B)** Western blot analysis of NCI-H322 cells treated as indicated (NCS at 2, 10, 50 ng/ml for 30 minutes; TAK-733, 2 hours).

Supplementary Figure 11

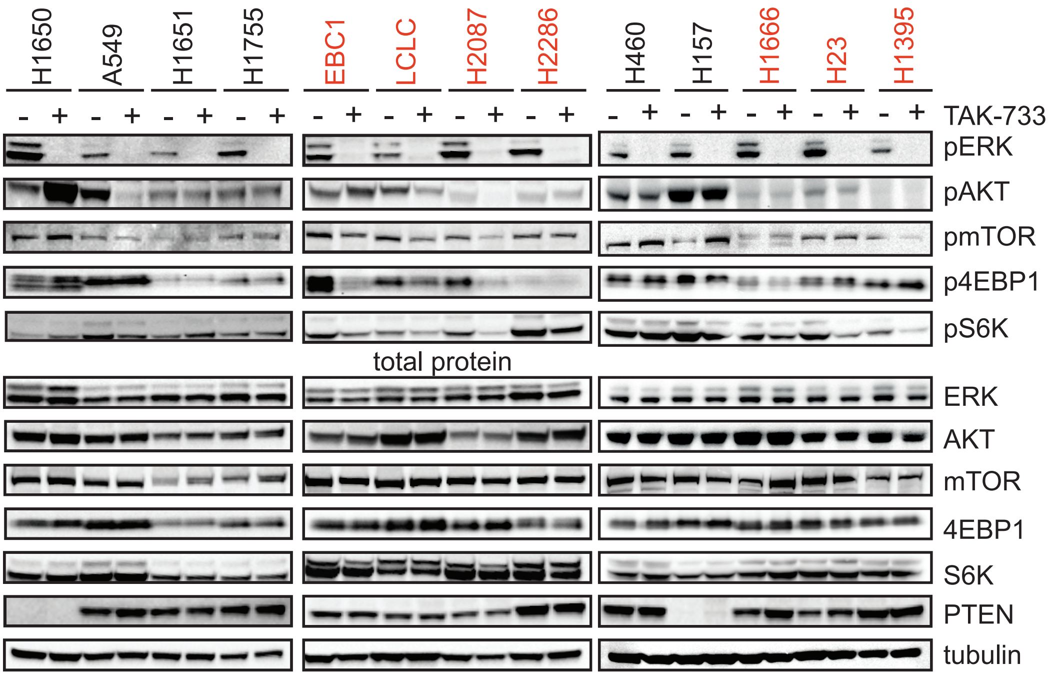

Supplementary Figure 11. Altered AKT/mTOR signaling in ATM mutant lung cancer cells. Western blot analysis (pERK (T202/204); pAKT (S473); p-mTOR (S2448); p4EBP1 (T37/46); pS6K (T389)) of indicated lung cancer cell lines treated with DMSO or 1 μM TAK-733 for 6 hours. ATM mutant cell lines are marked in red.

Supplementary Figure 12

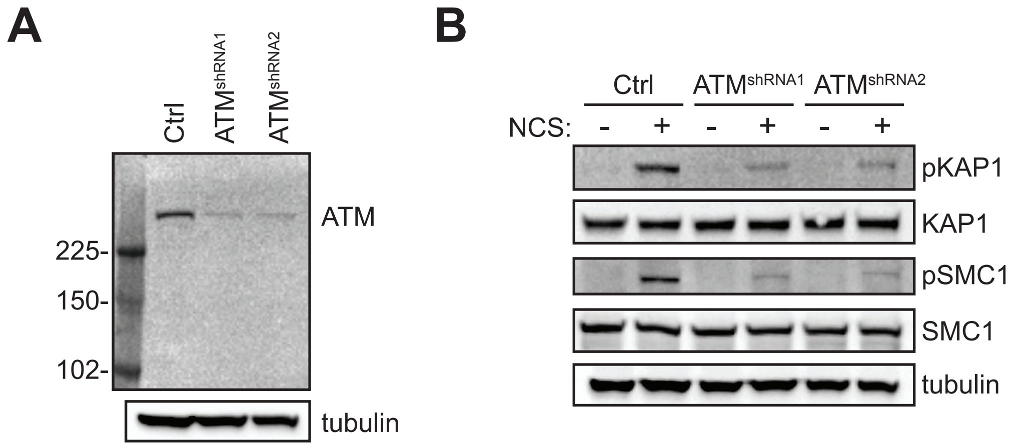

Supplementary Figure 12. Impaired DNA damage response in ATM knockdown NCI-H460 cells. (A) Western blot analysis of ATM expression in NCI-H460 cells infected with ATM knockdown vectors. **(B)** Western blot analysis of ATM kinase activity in NCI-H460 cells infected with indicated vectors. Phosphorylation of KAP1 and SMC1 in response to treatment with neocarzinostatin (50 ng/ml, 30 minutes) is shown.

Supplementary Figure 13 (I)

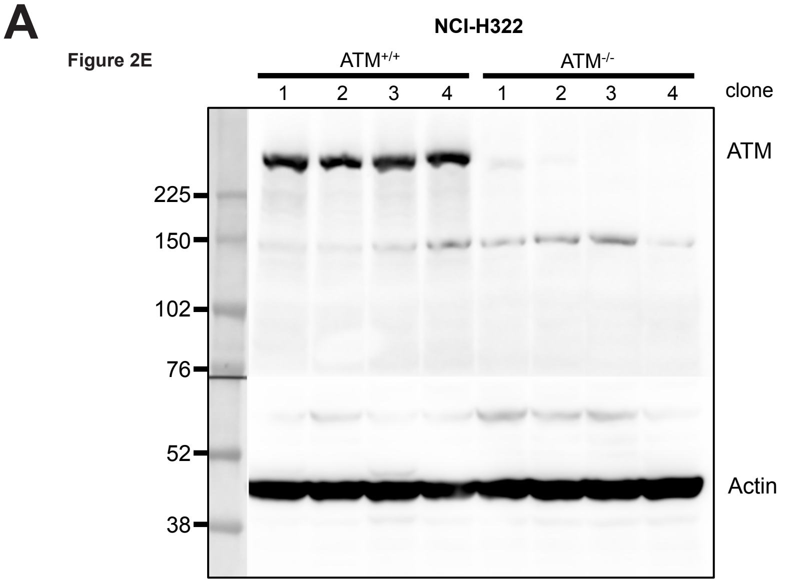

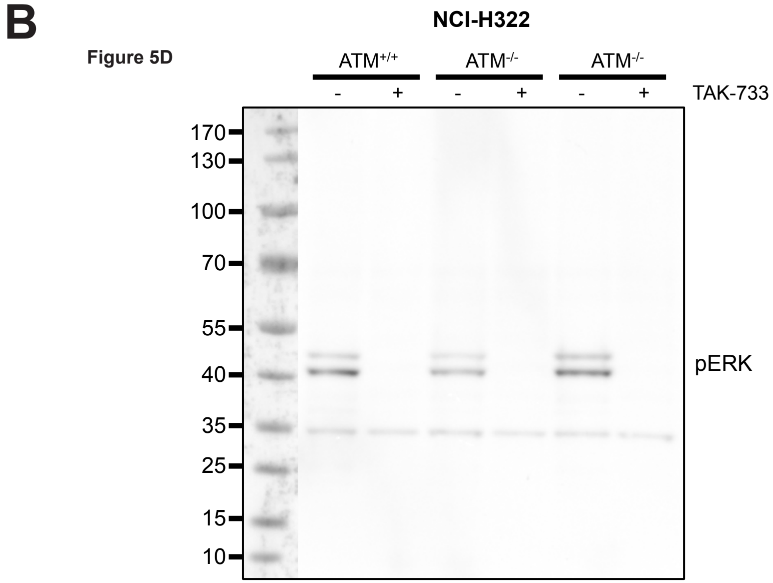

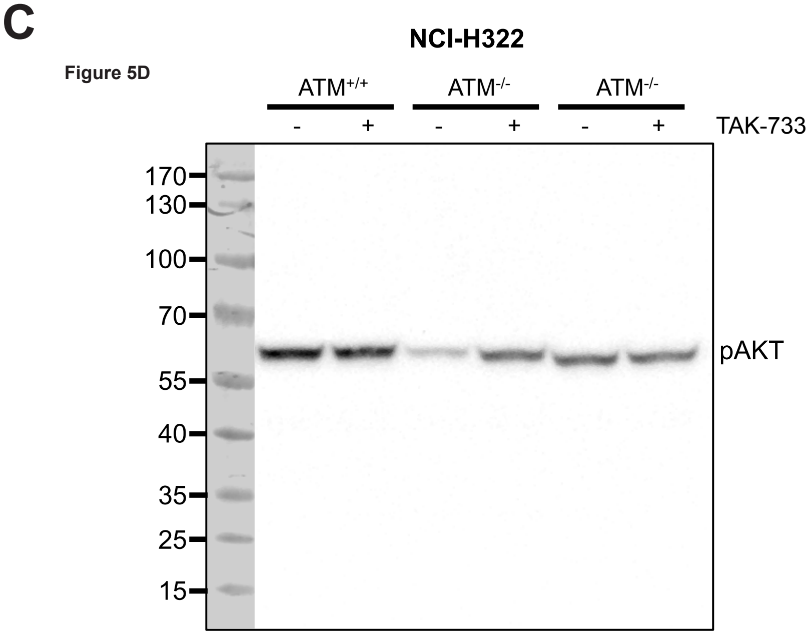

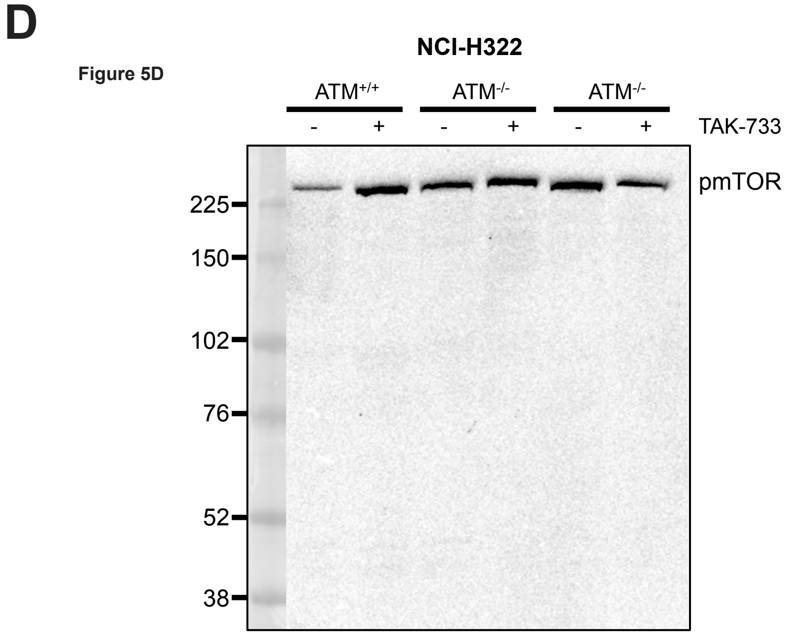

Supplementary Figure 13 (II)

E

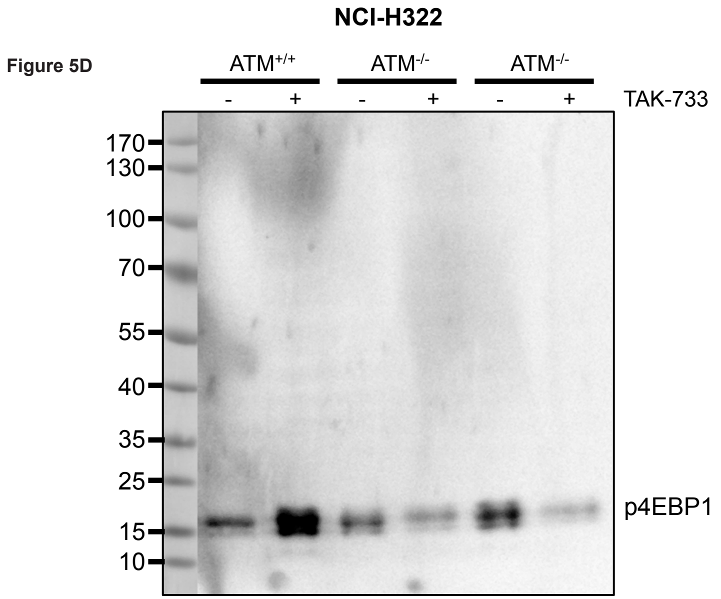

F

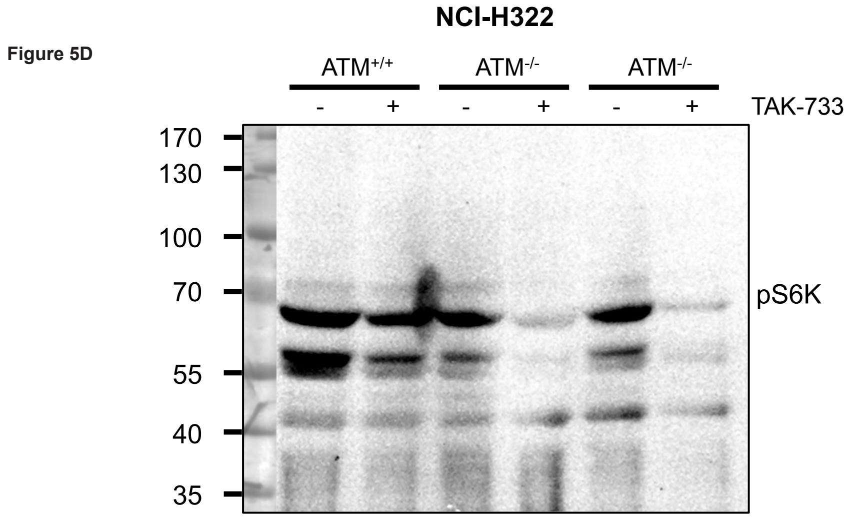

G

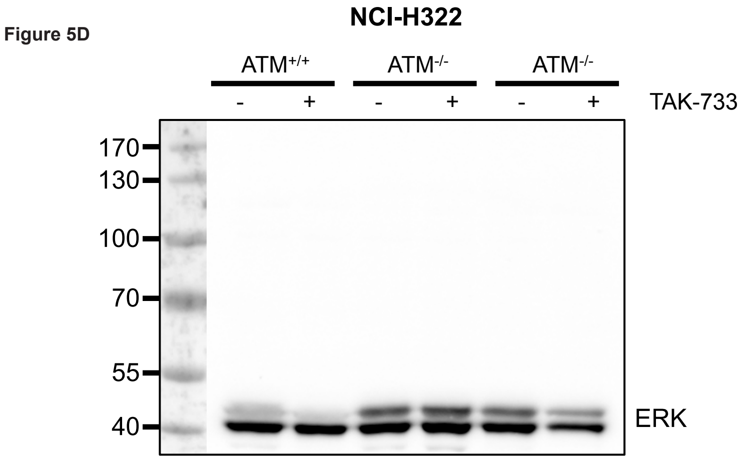

H

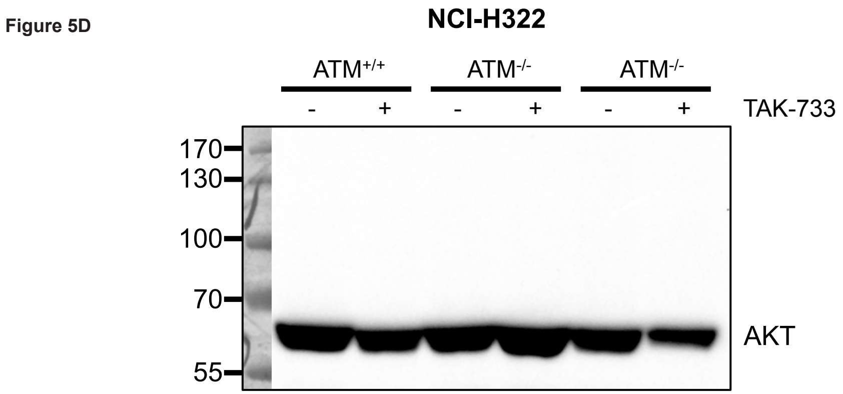

Supplementary Figure 13 (III)

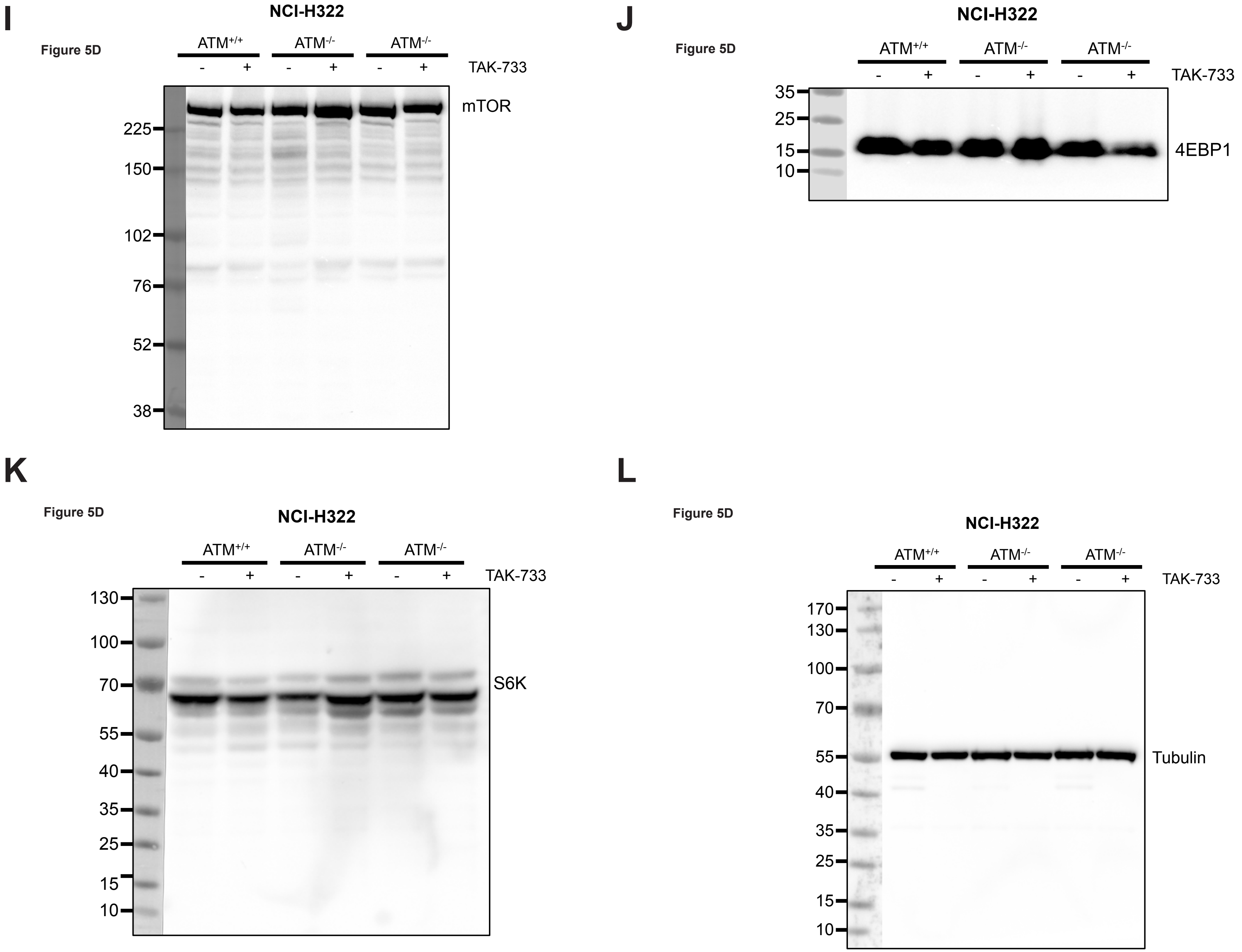

Supplementary Table 1

Constructs for the isogenic cell line panel

Gene	Vector designation	Sequence	Source
APC	pLKO.1-shAPC	CCGGTAATGAACACTACAGATAGAACTCGAGTTCTATCTGTAGTGTTCATTATTTTTG	TRCN0000010297
ATM	pLKO.1-shATM1	CCGGGGATTTGCGTATTACTCAGTTCAAGAGACTGAGTAATACGCAAATCCTTTTTT	NA
ATM	pLKO.1-shATM2	CCGGGACTTTGGCTGTCAACTTTCGTTCAAGAGACGAAAGTTGACAGCCAAAGTCTTTTTT	NA
BRG1 (SMARCA4)	pLKO.1-shBRG1	CCGGCATGCACCAGATGCACAAGTTCAAGAGACTTGTGCATCTGGTGCATGTTTTTT	designed from http://www.ncbi.nlm.nih.gov/pubmed/19149898
ERBB4	pLKO.1-shERBB4	CCGGCCAGACTACCTGCAGGAGTACCTCGAGGTACTCCTGCAGGTAGTCTGGTTTTTT	designed from http://www.ncbi.nlm.nih.gov/pubmed/17521571
NF1	pLKO.1-shNF1	CCGGGGACACAATGAGATTAGATTTCAAGAGAATCTAATCTCATTGTGTCCTTTTTT	NA
p14-ARF (CDKN2A)	pLKO.1-sh-p14ARF	CCGGCATGGTGCGCAGGTTCTTGTTCAAGAGACAAGAACCTGCGCACCATGTTTTTT	designed from http://www.ncbi.nlm.nih.gov/pubmed/14585358
PRKDC	pLKO.1-shPRKDC	CCGGGATCGCACCTTACTCTGTTCTCGAGAACAGAGTAAGGTGCGATCTTTTTT	designed from http://www.ncbi.nlm.nih.gov/pubmed/12438223
PTEN	pLKO.1-shPTEN	CCGGAAGGCACAAGAGGCCCTAGATTTCTCGAGAAATCTAGGGCCTCTTGTGCCTTTTTT	designed from http://www.ncbi.nlm.nih.gov/pubmed/17300726
SMAD4	pLKO.1-shSMAD4	CCGGGCAGACAGAAACTGGATTAAACTCGAGTTTAATCCAGTTTCTGTCTGCTTTTTG	TRCN0000040028
STK11	pLKO.1-shSTK11	CCGGGAAGAAGAAGTTGCGAAGGATCTCGAGATCCTTCGCAACTTCTTCTTCTTTTTG	TRCN0000000410

CRISPR sgRNA sequences

Gene	Targeted region	Sequence
ATM	Exon 6	GGATGCTGTTCTCAGACTGACGG
ATM	H23 mutation (c.5756)	ACTACATGAGAAGACCAAAGAGG

Supplementary Table 1. List of constructs used in the paper, including vectors, sequences and their original source.

Supplementary Table 2

DRUG NAME	TARGET	SCREENED CONCENTRATION (uM)		
(s)BI-2536 TsOH Salt	PLK1	1	3.8	
ABT-737	BCL2	2.5		
ABT-869	VEGFR	20		
AC220	FLT3	3.6		
AG13958	VEGF	1.7		
Akt-I-1	AKT1	50		
Akt-I-1,2	AKT1	20		
AMG-Tie2-1	TIE-2	1.7		
AS-252424	PI3K	6.6		
AT7519	CDK	1.3	5.2	
AT9283	Aurora Kinase	0.05		
AV-412	EGFR	0.02		
AV-951	VEGFR	4.4		
Axitinib	VEGFR	0.05		
AZ-960	JAK2	0.06		
AZD6244	MEK	0.17		
AZD7762	CHK	0.02	0.06	
BEZ235	PI3K/mTOR	0.01	0.04	
BI-D1870	p90RSK	0.05	0.2	
BMS-2	MET	4.4	18	
Bosutinib	SRC	0.02		
BX795	PDK1/TBK1	1.4		
BX912	PDK1	0.04		
CC-401	JNK	18.8		
CI-1033	EGFR/HER2	0.04		
CI-1040	MEK	0.17		
Cisplatin	DNA-crosslinking	2		
CP690550	JAK2	64		
CP-724714	HER2	4.3		
Crizotinib	ALK, c-MET	0.5		
CYC-116	Aurora Kinase	0.05	0.2	
CYT11387	JAK2	0.2		
Dasatinib	BCR/ABL	0.001	0.004	
Docetaxel	Microtubule	0.00005		
Doxorubicin	Topoisomerase II	0.005	0.01	
E7080	VEGFR	4.7		
Erlotinib	EGFR	0.1		
Etoposide	Topoisomerase II	0.05	0.1	
Flavopiridol	CDK	0.005		
GDC-0941	PI3K	0.04		
Gefitinib	EGFR	0.2		
Gemcitabine	Antimetabolite	0.001	0.002	
GSK690693	AKT1	1.2	4.7	
GW441756	TRKA	7	29	
IC87114	PI3K	5	20	
Ifosfamide	Nitrogen mustard	50		
Imatinib-Mesylate	BCR-ABL	3.4		
Irinotecan	Topoisomerase I	0.2		
JNJ-38877605	cMET	20		
JNJ-7706621	CDK/Aurora Kinase	0.2		
Ki20227	VEGFR/cFMS	4.2	17	
KU0063794	mTOR	0.2		
KU55933	ATM	5		

DRUG NAME	TARGET	SCREENED CONCENTRATION (uM)		
Lapatinib	HER2	0.14		
Masatinib	cKIT	0.16		
Merck-5	JAK	0.26		
Motesanib	VEGFR	5.4	20	
MP-470	Multi Kinase	45		
Nilotinib	BCR-ABL/KIT	1.5		
Paclitaxel	Microtubule	0.0002		
Pazopanib	VEGFR	1.8		
PD0325901	MEK	0.17		
PD173955	BCR-ABL	1.1		
PD173955-Analogue 1	BCR-ABL	1.8		
Pemetrexed	Antifolate, antimetabolite	1.5		
PF-04217903	cMET	20		
PF431396	PYK2	0.02		
PF-562271	FAK/PYK2	0.16		
PI103	PI3K	0.23		
PIK-75	PI3K	0.001		
PIK-90	PI3K	0.23		
PIK-93	PI3K	2		
PLX4720	BRAF	5	20	
Purvalanol B	PARP	1.2		
R1487	MAPK	47		
Rho-15	ROCK	1.2	5	20
RWJ-67657	MAPK	4.7		
SB202190	MAPK	6	24	
SB203580	MAPK	20		
SB216763	GSK3	1.2	5	20
SB242235	MAPK	23		
SB590885	BRAF	18		
SNS-032	CDK	0.005	0.02	
SNS-314	Aurora Kinase	1.2	20	
Sorafenib	Multi Kinase	0.17		
SR3677	ROCK	4.9		
SU-5402	FGFR1	6.8	27	
SU-6668	Multi Kinase	26		
Sunitinib	Multi Kinase	1.5		
TAK-715	MAPK	5		
Tandutinib	FLT3	0.9	3.6	
TG100115	PI3K	23		
TG101209	JAK2	0.16		
TG101209 Deriv 1	JAK2	0.16		
TG101209 Deriv 2	JAK2	0.16		
TG101348	JAK2	0.38		
TGX221	PI3K	55		
Topotecan	Topoisomerase I	0.001		
Vandetanib	VEGFR	0.17		
Vargatef	VEGFR/PDGFR	0.9		
Vinblastine	Microtubule	0.0005		
Vinorelbine	Anti-mitotic	0.001		
VX-680	Aurora Kinase	0.04	0.17	
VX702	MAPK	50		
YM201636	PI3K	1.6		
ZSTK474	PI3K	0.05	0.2	

Supplementary Table 2. List of small molecule compounds with their primary targets and concentrations used in the screen.

Supplementary Table 3

CRISPR-Cas9 editing of H322 lung cancer cell line

NCI-H322		
Total clones analyzed		59
Clones edited		12
	In frame	1
	Heterozygous frame shift	7
	Homozygous frame shift	4

Clones	Allele 1	Allele 2
WT	GATGCTGTTCTCAGACTGACGGATT	GATGCTGTTCTCAGACTGACGGATT
1	GATGCTGTTCTC----TGACGGATT	GATGCTGTTCTC----TGACGGATT
2	GATGCTGTTCTCAGACCTGACGGATT	(del. >100bp)GACGGATT
3	GATGCTGTTCTCA----GACGGATT	GATGCTGTTCTCA----GACGGATT
4	GATGCTGTTCTC----TGACGGATT	GATGCTGTTCTCAGAC-GACGGATT

Supplementary Table 3. Overview of isolated clones edited with CRISPR-Cas9 system. Numbers of individual clones modified by heterozygous and homozygous frame shift is depicted. Edited sequence for both alleles is shown for the homozygously edited clones and compared to wild type.

Supplementary Table 4

Off target site prediction and analysis

sgRNA ATM exon 6	Gene	Mis matches	PAM	Clones with mutation (Sanger)	CRISPR.mit.edu score
GGATGCTGTTCTCAGACTGA CGG	ATM	NA	Yes	NA	NA
AGAAGCAGTTCTCAGACTGA AAG	TCP1	3	No	0/2	1.7
GTATGTTCTCCTCAGACTGA GGG	GAPDH	4	Yes	0/2	0.8
TGACGCTTTTCTCAGACTGC CAG	GALNT2	4	No	0/2	0.7
GGAAGCAGAGCTCAGACTGA AGG	VAV1	4	Yes	0/2	0.5
GCATTATGTTATCAGACTGA TAG	CLEC9A	4	No	0/2	0.5
**sgRNA ATM H23 mutation**	**Gene**	**Mis matches**		**Clones with mutation (Sanger)**	**CRISPR.mit.edu score**
ACTACATGAGAAGACCAAAG AGG	ATM (H23)	NA	Yes	Verified in NCI-H23	NA
ACTACATGAGAAGACAAAAG AGG	ATM (WT)	1	Yes	NA	17.2
GCTAGATGAAATGACCAAAG AAG	RPGR	4	No	NA	0.7
AGTGGATGAGAAGACCAAGG AAG	TLN1	4	No	NA	0.5

Supplementary Table 4. CRISPR off-target site prediction and analysis. Off-target sites for individual sgRNAs were predicted using the analysis tool available at crispr.mit.edu. Predicted off-target score is depicted in the rightmost column. Two clones were analyzed by Sanger sequencing for the presence of off-target modification predicted for sgRNA exon 6. No editing was observed within 5 top predicted off-target sites.

Supplementary Table 5

	Sensitivity*	True positive	False positive
KRAS/BRAF	**9/14 (64%)**	9/39 (23%)	30/39 (76%)
ATM	4/14 (29%)	**4/7 (57%)**	**3/7 (43%)**
KRAS/BRAF/ATM	**11/14 (78%)**	**11/41 (27%)**	**28/41 (68%)**

*Activity area >2 is considered MEKi sensitive

Supplementary Table 5. Predictive value of ATM mutation as a biomarker for MEKi response. Calculated sensitivity, false positive rate and true positive rate for ATM, KRAS/BRAF and ATM+KRAS/BRAF based on the CCLE cell line sensitivity data.
